# Effects of Poly(cyclohexanedimethylene terephthalate) on Microstructures, Crystallization Behavior and Properties of the Poly(ester ether) Elastomers

**DOI:** 10.3390/ma10070694

**Published:** 2017-06-24

**Authors:** Yi-Cheng Feng, Hui Zhao, Tong-Hui Hao, Guo-Hua Hu, Tao Jiang, Qun-Chao Zhang

**Affiliations:** 1 Hubei Collaborative Innovation Center for Advanced Organic Chemical Materials, Ministry of Education Key Laboratory of Green Preparation and Application for Functional Materials, School of Materials Science & Engineering, Hubei University, Wuhan 430062, China; fengyicheng0221@icloud.com (Y.-C.F.); zhh0625@foxmail.com (H.Z.); haoth@hubu.edu.cn (T.-H.H.); jiangtao@hubu.edu.cn (T.J.); 2 Laboratory of Reactions and Process Engineering, CNRS-University of Lorraine, 1 rue Grandville, BP 20451, 54001 Nancy Cedex, France; guo-hua.hu@univ-lorraine.fr

**Keywords:** poly(ester ether), copolymerization, microstructures, crystallization behavior

## Abstract

To understand the role of molecular structure on the crystallization behavior of copolyester in thermoplastic poly(ether ester) elastomers (TPEEs), series of poly(butylene-co-1,4-cyclohexanedimethylene terephthalate) (P(BT-co-CT))-b-poly(tetramethylene glycol) (PTMG) are synthesized through molten polycondensation process. The effects of poly(cyclohexanedimethylene terephthalate) (PCT) content on the copolymer are investigated by Fourier transform infrared spectroscopy (FT-IR), ^1^H and ^13^C nuclear magnetic resonance (NMR), gel permeation chromatographs (GPC), wide-angle X-ray diffraction (WAXD), differential scanning calorimetry (DSC), thermogravimetric analysis (TGA), mechanical, and visible light transmittance tests. FT-IR and NMR results confirm the incorporation of PCT onto the copolymer. WAXD and DSC indicate that the crystalline structure of the copolymers changed from α-PBT lattice to trans-PCT lattice when the molar fraction of PCT (*M*_PCT_) is above 30%, while both crystallization and melting temperatures reach the minima. An increase in *M*_PCT_ led to an increase in the number sequence length of PCT, the thermal stability and the visible light transmittance of the copolymer, but to a slight decrease in tensile strength and elastic modulus.

## 1. Introduction

Thermoplastic poly(ether ester) elastomers (TPEEs) are usually block copolymers composed of polyester segment (hard segment) and polyether segment (soft segment) [1,2,3]. The hard segment forms physical crosslinks, while the soft segment is highly elastic and contributes to flexibility. Thus, in addition to possessing rubber-like properties, TPEEs can also be processed similar to thermoplastics. Because of the unique processing and mechanical properties, TPEEs are considered to be “third-generation synthetic rubbers”, which are applied in numerous applications such as electronics and automotive industries, in skin tissue engineering, and so on [4,5,6,7].

Poly(butylene terephthalate) (PBT)-b-poly(tetramethylene glycol) (PTMG) is one of the most typical TPEEs with useful properties such as high tensile strength and elastic modulus. This is due to the thermodynamic immiscibility between the hard and soft segments, which dictates the crystallization behavior, morphology, and thermal and mechanical properties of PBT-b-PTMG [8,9]. Many attempts have been made to improve its properties. Celebi et al. attempted to add TiO_2_ or ZnO particles to the PBT-b-PTMG to improve its thermal stability, storage modulus, and mechanical properties [10]. Zhang et al. partially substituted aromatic segment PBT with aliphatic poly(butylene succinate) (PBS) to accelerate the degradation rate [11]. Liu et al. incorporated an aliphatic ring to partially replace the aromatic ring in PBT-b-PTMG to decrease its melting temperature without altering thermal stability [12].

However, the hard segment, PBT, is easy crystallized, which limits the transparency of TPEEs. Hence, there is still a need to improve properties of TPEEs with the emphasis on their transparency. The key to obtain this property is how to synchronize the mechanical properties with crystallization property such as degree of crystallinity, crystallization rate and crystallization temperature. Copolymerization is a powerful method to combine the advantages of corresponding homopolymers [13,14]. At the same time, it is also an effective way to decrease the average sequence length of the segments, as well as the degree of crystallinity [15].

It is worth noting poly(ethylene glycol-co-1,4-cyclohexanedimethanol terephthalate) (PETG) copolyester, which shows special crystallization behavior and high transparency [16,17,18]. PETG is prepared by partially replacing the ethylene glycol (EG) groups of poly(ethylene terephthalate) (PET) with 1,4-cyclohexanedimethanol (CHDM) groups. The PETG copolymer is essentially amorphous when CHDM content is in the range of 32–62%, but its mechanical properties are close to those of PET [19]. CHDM has two isomers, *trans* (axial–axial or equatorial–equatorial) and *cis* (axial–equatorial), the former being more stable than the latter. Many investigations on the copolymers with cyclohexanedimethylene group reveal that the cyclohexane ring in the structure improves the thermal stability, mechanical properties and the transparency of the copolymer [20,21,22,23,24]. Therefore, the use of CHDM as a co-monomer makes the development of new types of optically interesting thermoplastic elastomers possible.

To improve the transparency of the copolymer, this work attempts to incorporate CHDM as a third co-monomer. The content of the hard segment PBT is decreased upon introducing PCT which has good crystallization behavior. The resulting copolymers are denoted as (PBT-co-PCT)-b-PTMG, which have constant soft segment length and molar fraction at 1000 g mol^−1^ and 20%, respectively. The microstructure, crystallization behavior and properties of the copolymer are affected by the copolyester hard segments. Furthermore, compared to traditional TPEEs, the (PBT-co-PCT)-b-PTMG copolymers synthesized in this work show better performance in many aspects, such as high transparency and good thermal stability. For the sake of simplicity, they are designated by their theoretical PCT molar fractions (*M*_PCT_) as PCT-*M*_PCT_.

## 2. Materials and Methods

### 2.1. Materials

Dimethyl terephthalate (DMT), 1,4-butanediol (BDO), catalysts tetrabutyl titanate (TBT) and magnesium acetate (MgAc_2_) were purchased from Sinopharm Chemical Reagent Co. Ltd. (Shanghai, China). CHDM was purchased from Aladdin Reagent Co. Ltd. (Shanghai, China); it was the mixture of 69% *trans* and 31% *cis*, and PTMG with an average molar weight of 1000 g mol^−1^ was supplied by BASF (Shanghai, China). The antioxidants (Irganox 1010 and Irgafos 168) were supplied by Ciba (Basel, Switzerland). All of these chemical materials were dried under vacuum at 80 °C for 24 h to a constant mass before use.

### 2.2. Experiments

(PBT-co-PCT)-b-PTMG copolymers were synthesized through molten polycondensation process in a dried steel reactor (Weihai Xingyu Chemical Machinery Co., Weihai, China) equipped with a stirrer, a condenser and a gas inlet.

A typical procedure for the synthesis of (PBT-co-PCT)-b-PTMG copolymers is shown by the example of PCT50. The following chemicals were charged to the reactor under nitrogen: co-monomers DMT (0.5 mol), CHDM (0.255 mol), BDO (0.445 mol) and PTMG (100 g); catalysts TBT (0.1% of the total mass) and MgAc_2_ (0.02% of the total mass); and antioxidants Irganox 1010 and Irgafos 168 (0.5% of the total mass). Then, the reactor was heated up to 205 °C at 5 °C min^−1^ and remained at that temperature for 3 h to reach the end of the transesterification. The polycondensation was then performed upon raising the temperature to 265 °C and the pressure was below 50 Pa. After 3.5 h, the copolymer was extruded from the reactor under nitrogen, cooled down in a water bath, and dried in a vacuum dryer at 50 °C for 24 h to a constant mass.

**Figure 1:**
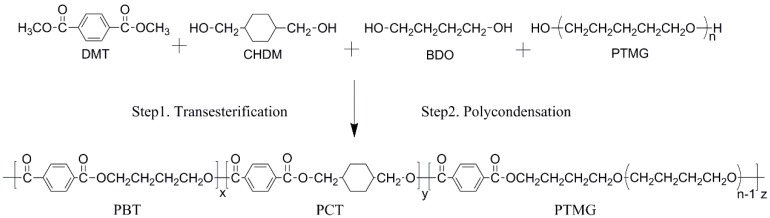
Synthesis route of the (PBT-co-PCT)-b-PTMG copolymers.

It is worth noting that the reaction time and temperature were increased slightly with the increasing CHDM content. The reactions involved in these syntheses were formulated in Figure 1 and the compositions of all samples are given in Table 1.

### 2.3. Characterization

ATR-FTIR spectroscopy was performed on a FTIR spectrometer in the attenuated total reflectance mode (PE-Spectrum one, Perkinelmer, Waltham, MA, USA). The spectra were scanned from 600 to 4000 cm^−1^ with a resolution of 4 cm^−1^.

^1^H NMR and ^13^C NMR spectra were recorded on a spectrometer of Inova-600, USA, operating at 600 MHz. They were used for determining the copolymer composition and average sequence length, respectively. Deuterated chloroform (CDCl_3_) was used as a solvent and tetramethylsilane (TMS) as an internal reference.

The number average molecular weight (*M*_n_) and the dispersity (*M*_w_/*M*_n_) were determined by GPC (PL-GPC220, Agilent Technologies, CA, USA). The GPC measurements were carried out at 150 °C using 1,2,4-trichlorobenzene as the eluent at a flow rate of 1 mL min^−1^.

Wide-angle X-ray diffraction patterns were recorded on a WAXD diffractometer of type Bruker D8 Advance (Bruker, Karlsruhe, Germany) in the 2θ range from 5° to 40° at a scanning rate of 2° min^−1^ for the copolymers prepared by a hot press. The crystal size in the perpendicular direction of the planes was calculated using the Scherrer’s formula [25]:

*L* = *K*λ/βcosθ (1)

where *L* is the crystal size; *K* is a structure constant, taken as 1; λ is the wavelength of the monochromatic X-ray beam (0.154 nm for CuK_α_ radiation); β is the full width at half maximum for the diffraction peak (rad); and the θ is the Bragg angle (°).

A differential scanning calorimeter of type Q-2000 (TA, Newcastle, PE, USA) was used to investigate the thermal properties of the copolymers. The measurement was performed using about 10 mg sample sealed in an aluminum pan under the nitrogen atmosphere. Firstly, the sample was heated to 300 °C rapidly and kept isothermally for 1 min to remove the previous thermal history. Secondly, the sample was quenched to −80 °C and heated to 300 °C at a speed of 10 °C min^−1^ to detect the glass transition temperature. Finally, a second cool scan and a third heat scan was performed at a speed of 10 °C min^−1^ to investigate the thermal properties of the copolymers. The crystallinity of hard segments was measured from the heat fusion obtained from DSC curves using Equation (2) according to group contribution theory [26,27],

(2) $$WC=\frac{\Delta H_{m}}{m_{PBT}\times \Delta H_{PBT}^{0}+m_{PCT}\times \Delta H_{PCT}^{0}}$$

where $\Delta H_{PBT}^{0}$ (144.5 J g^−1^) [28] and $\Delta H_{PCT}^{0}$ (102.0 J g^−1^) [29] are the heats of fusion of 1 mol PBT and PCT perfect crystals, respectively; and the mass fractions of the two types of hard segments are designated as *m_PBT_* and *m_PCT_*, respectively.

The thermal stability of the copolymers was evaluated by TGA of type TASDT-Q600 (TA, Newcastle, PE, USA). The measurement was performed under nitrogen and oxygen flow, respectively. The heating rate was 20 °C min^−1^ in range from 30 to 800 °C.

The specimens for tensile testing were prepared by a hot press with the following dimensions: 33 × 6.2 × 2 mm^3^. Stress-strain curves were recorded by a CMT4104 testing machine (SUNS, Shenzhen, China) at a constant crosshead speed of 50 mm min^−1^, according to the standard ISO 37-2011. At least five specimens were tested and the average values of the elastic modulus (*E*), tensile strength (σ_max_) and elongation at break (ε*_b_*) were calculated.

The specimens for optical testing were prepared by a hot press and their thickness was 2 mm. The visible light transmittance and haze were recorded on a Transmittance haze tester (WGT-S, Shanghai, China) according to ISO 14782.

## 3. Results and Discussion

### 3.1. Structure and Molecular Weight Analysis

**Figure 2:**
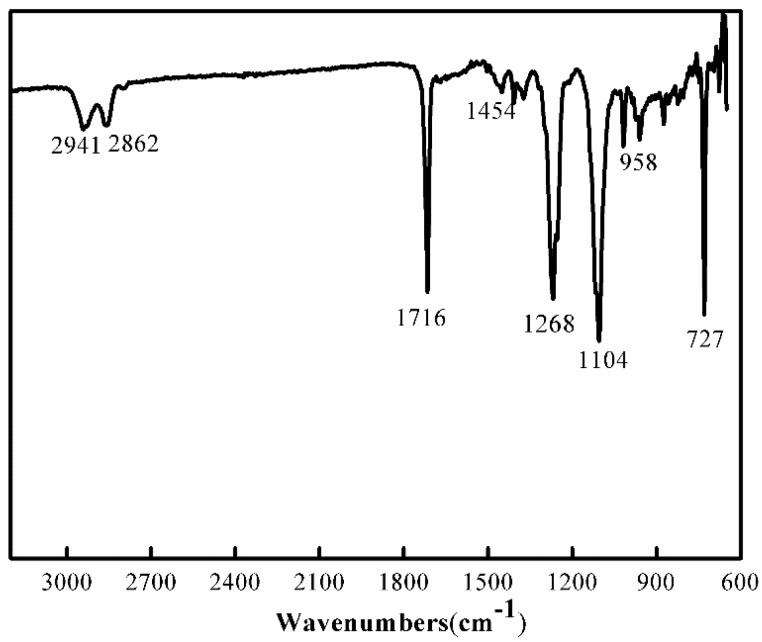
ATR-FTIR spectra of the sample PCT50.

The structure of those copolymers is first probed by a FTIR spectrometer in an ATR mode. Figure 2 shows the spectrum of PCT50 as an example. The peaks at 2941 and 2862 cm^−1^ are assigned to the asymmetric and symmetric aliphatic C–H stretching vibrations of PBT [10]. C=O stretching peak and ester group skeleton peak are observed at 1715 and 1268 cm^−1^, respectively. The C–O–C skeleton asymmetric vibration of soft segment, CH_2_ bending vibration, C–H stretching vibration of cyclohexylene ring and C–H vibration of benzene ring appear at 1104, 1454, 958 and 727 cm^−1^, respectively [30].

**Figure 3:**
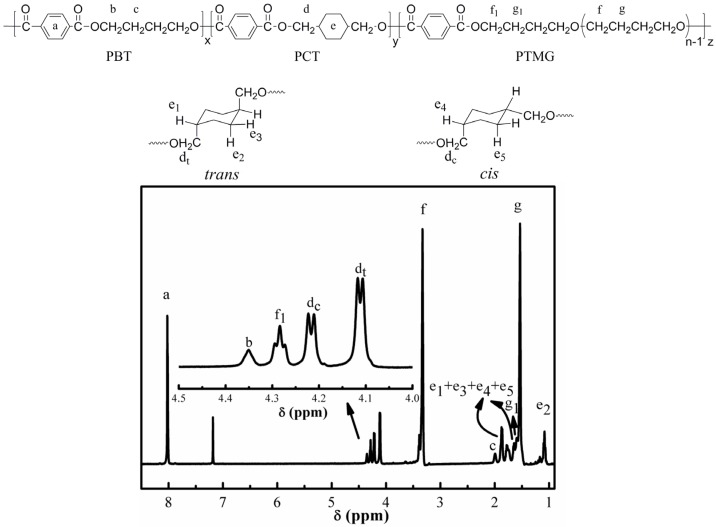
^1^H nuclear magnetic resonance spectra of sample PCT50.

Figure 3 shows a typical ^1^H NMR spectrum of PCT50, which corroborates its ATR-FTIR spectrum. Peak a at 8.01 ppm is the characteristic peak of protons in the benzene ring and stands for the total molar content of the acidic moieties herein. Peaks b and c, with chemical shifts at 4.35 and 1.98 ppm, respectively, correspond to the methylene protons of the hard segments PBT. The splits with chemical shift at 4.21 and 4.11 ppm are ascribed to the resonance peaks of methylene protons in CHDM moieties with *cis* and *trans* conformation and marked as *d_c_* and *d_t_*, respectively. The proton resonances of the cyclohexylene group appear between 1.08 ppm and 1.86 ppm and are marked as e_1_, e_2_, e_3_, e_4_ and e_5_, respectively. Peak f at 3.40 ppm and g at 1.53 ppm correspond to the soft segment. The chemical shifts of the tetramethylene protons attached to the ester group of the soft segment are peaks f_1_ at 4.36 ppm and g_1_ at 1.59 ppm [31,32].

Based on the ^1^H NMR spectra, the copolymer compositions can be determined from the respective peak areas as follows:

(3) $$M_{\mathrm{PBT}}=\frac{I_{b}}{I_{a}}$$

(4) $$M_{\mathrm{PCT}}=\frac{I_{d_{c}}+I_{d_{t}}}{I_{a}}$$

(5) $$M_{\mathrm{PTMG}}=\frac{I_{f_{1}}}{I_{a}}$$

where *M*_PBT_, *M*_PCT_ and *M*_PTMG_ denote the molar fractions of butylene terephthalate (BT), 1,4-cyclohexanedimethanol terephthalate (CT) and PTMG, respectively. *I_a_*, *I_b_*, *I_dc_*, *I_dt_* and *I_f1_* represent the integral areas of peaks *a*, *b*, *d_c_*, *d_t_* and *f_1_* in Figure 3, respectively. Table 2 shows that the compositions calculated by Equations (3)–(5) match relatively well the feed compositions.

The theoretical *M*_PTMG_ is fixed at 20% and the measured values are very close to it. However, the measured values of *M*_PCT_ are always slightly higher than the theoretical ones. This is because CHDM is more difficult to remove than BDO during the polycondensation under the vacuum. Moreover, BDO participates in side reactions during the transesterification upon forming tetrahydrofuran. It is also interesting to note that the molar ratio of *trans* to *cis* isomers of the CT is almost constant at about 2.3 to 1 for all copolymers. This indicates that isomerization between *trans* and *cis* of CHDM did not occur during the polymerization.

Table 2 also shows the molecular weights of the copolymers. Both the number and weight average molecular weights of the copolymers decrease very slightly with increasing CT content. This is because it is more difficult to remove CHDM than BDO during the polycondensation under the vacuum.

### 3.2. Sequence Distribution Analysis

**Figure 4:**
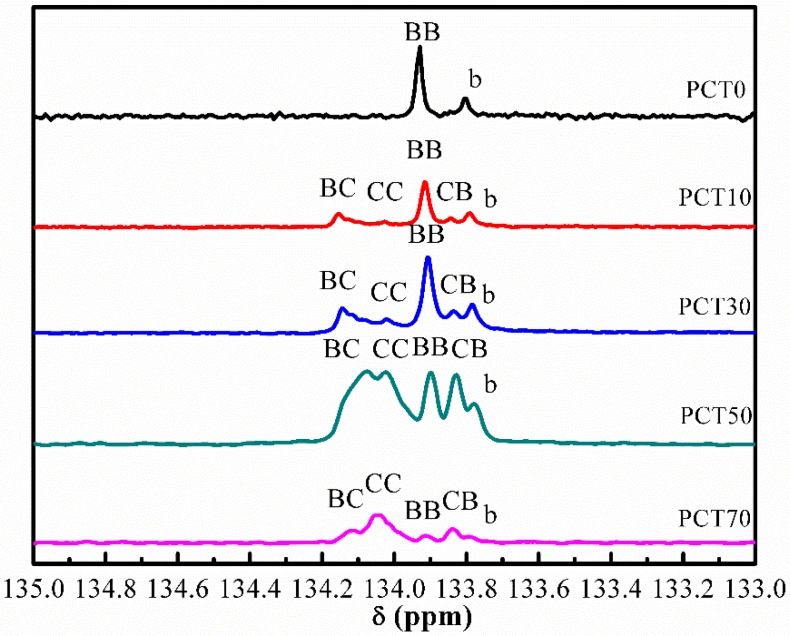
^13^C NMR spectra of the (PBT-co-PCT)-b-PTMG copolymers.

Since Yamadera and Murano [33] proposed a method to determine the average sequence length and the distribution of copolyesters, many studies focused on the analysis of the microstructures of copolymers by ^13^C NMR [34,35,36]. Figure 4 shows the ^13^C NMR spectra of the (PBT-co-PCT)-b-PTMG copolymers. The quaternary carbon atom resonance in benzene rings splits into five peaks. Peak b is due to the quaternary carbon atom of the benzene ring linked to the soft segment PTMG because its relative integral intensity changes slightly with increasing *M*_PCT_ at a constant molar fraction of soft segment. The sequence distribution of the two hard segments, PBT and PCT, is analyzed according to the following procedure.

The molar fractions of *BT* moiety and *CT* moiety are determined from integration of the peaks. *P_B_* (*P_C_*) is the molar fraction of the *BT* (*CT*) moiety, and *f_BB_*, *f_BC_*, *f_CB_* and *f_CC_* correspond to the proportions of the integrated intensities of four types of dyads, *BB*, *BC*, *CB* and *CC*, respectively. Then, *P_BC_* (*P_CB_*) is the probability of finding a *BT* (*CT*) moiety next to a *CT* (*BT*) moiety. The number average sequence lengths of *BT* and *CT* moieties are designed by *L_nB_* and *L_nC_*, respectively. The degree of randomness is designated by *DR* and is equal to 0 for a mixture of homopolymers, and is equal to 2 for an alternating distribution [37,38]. The above parameters can be calculated by the following equations:

(6) $$P_{B}=\frac{f_{BC}+f_{CB}}{2}+f_{BB}\;P_{C}=\frac{f_{BC}+f_{CB}}{2}+f_{CC}$$

(7) $$P_{BC}=\frac{f_{BC}+f_{CB}}{2P_{B}}\;P_{CB}=\frac{f_{BC}+f_{CB}}{2P_{C}}$$

(8) $$L_{\mathrm{nB}}=\frac{2P_{B}}{f_{BC}+f_{CB}}\;L_{\mathrm{nC}}=\frac{2P_{C}}{f_{BC}+f_{CB}}$$

(9) $$DR=p_{BC}+P_{CB}$$

Their values are gathered in Table 3. All the values of *DR* are very close to 1, indicating that the sequence distribution in the copolymers is random in the entire range of the *CT* composition. The number average sequence length *L_nB_* decreases while *L_nC_* increases with increasing *CT* content, as expected. This is due to the increasing content of monomer CHDM, which leads to an increase of the probability that the bis(hydroxymethylcyclohexane)terephthalate (BHCT) reacts with BHCT during the polycondensation reaction.

### 3.3. WAXD Analysis

**Figure 5:**
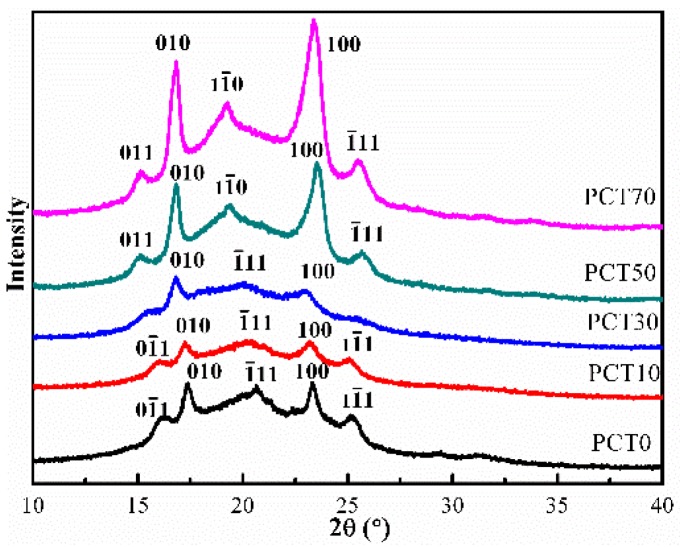
WAXD patterns of the (PBT-co-PCT)-b-PTMG copolymers.

Figure 5 shows the WAXD diagrams of the (PBT-co-PCT)-b-PTMG copolymers. The intensities of the peaks increase with the increasing PCT content. Table 4 shows the corresponding WAXD data.

According to Briber and Thomas [39], hard segments PBT moieties in a thermoplastic elastomer always organize in the α-PBT form when crystallized from the molten state, and the lattice is independent of the concentration of soft segment. Therefore, for the (PBT-co-PCT)-b-PTMG copolymers when *M*_PCT_ is below 30%, the hard segments are organized in the form of α-PBT crystal. The peaks (and the corresponding Miller indices) of the α-PBT at 2θ are 16.12° (0$\bar{1}$1), 17.38° (010), 20.91° ($\bar{1}$01), 23.36° (100), and 25.31° (1$\bar{1}$1). The unit cell of α-PBT is triclinic with the cell parameters *a* = 0.483 nm, *b* = 0.594 nm, *c* = 1.159 nm and α = 99.7°, β = 115.2°, and γ = 110.8°, according to the reported literature [40].

Boye [41] reported that WAXD pattern of PCT homopolymer with a *trans*/*cis* isomer ratio of more than 68/32 was the same as the pure *trans*-PCT. In this study, *trans*/*cis* isomer ratio is around to 70/30, as shown in Table 1. Since the *trans*/*cis* isomer ratios of the (PBT-co-PCT)-b-PTMG copolymers are about 70/30, their CT moieties for *M*_PCT_ of more than 30% are expected to form triclinic crystal lattices with the reported cell parameters: *a* = 0.637 nm, *b* = 0.663 nm, and *c* = 1.42 nm; and α = 89.35°, β = 47.11°, and γ = 134.36°. The peaks (and the corresponding Miller indices of *trans*-PCT) at 2θ are 14.94° (011), 16.68° ($1\bar{1}1$), 19.32° ($1\bar{1}0$), 23.26° (100), and 25.57° ($\bar{1}11$), according to the reported literature [32].

Figure 5 shows that when *M*_PCT_ is less than 30%, the CT moieties do not intervene in the formation of α-PBT crystal lattices. When it is above 30%, the intensities of the peaks corresponding to the *trans*-PCT crystal lattices increase rapidly with increasing *M*_PCT_ because CT moieties with an average sequence length exceeding 1.5 can crystallize. The transition from α-PBT crystal lattices to *trans*-PCT crystal lattices of the (PBT-co-PCT)-b-PTMG copolymers can be explained from the viewpoint of cohesive energy (E).

Cohesive energy (E) is used to describe the energetic interactions between the segments of each component polymer molecules in the molten state. The cohesive energies corresponding to A and B components in an A/B binary random copolymer are assumed to be proportional to the copolymer composition and can be calculated according to the group contribution method [29,31]. When the cohesive energies of A and B components are identical, both A and B can co-crystallize into a crystal lattice at certain compositions. According to the group contribution method, the cohesive energies of hard segments PBT and PCT are 89,420 J mol^−1^ and 119,160 J mol^−1^ [15], respectively. Moreover, the PCT composition of the (PBT-co-PCT)-b-PTMG copolymer at which the two hard segments PBT and PCT undergo co-crystallization, denoted as M^*^_PCT_, is 43%. This value is close to the molar fraction of the CT hard segments in Table 1. That is to say, theoretically, when *M*_PCT_ is less than M^*^_PCT_, only BT moieties can be crystallize and form α-PBT crystal lattices, and the CT moieties would be repelled into non-crystallized regions; and, when *M*_PCT_ is above M^*^_PCT_, CT moieties begin to crystallize and form *trans*-PCT crystal lattices, while the BT moieties would be repelled into non-crystallized regions. It is worth noting that the crystal lattice transition from α-PBT to *trans*-PCT also manifests itself by the minimum melting temperature, minimum heat of fusion, and the discontinuous jump of the glass transition temperature for the (PBT-co-PCT)-b-PTMG copolymer, as will be shown below.

In Table 4, the crystal size of the α–PBT crystals of PCT0 are larger than those of PCT10 and PCT30 under the same crystallization condition, and those of the *trans*-PCT crystals of PCT70 are larger than those of PCT50. These results indicate that the crystal size of the (PBT-co-PCT)-b-PTMG copolymer decreases first and then increases with the increasing CT content.

### 3.4. DSC Analysis

**Figure 6:**
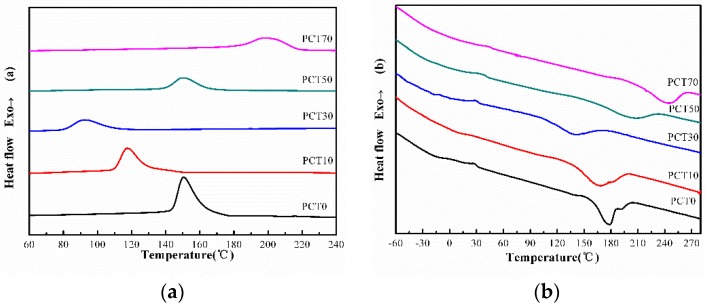

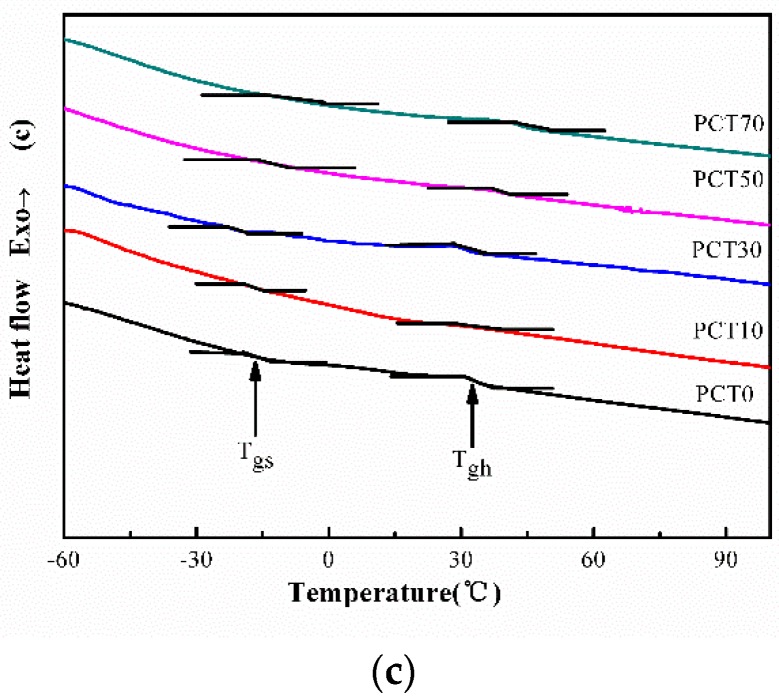
DSC traces of the (PBT-co-PCT)-b-PTMG copolymers at 10 °C min^–^^1^ in nitrogen: (**a**) cooling traces; (**b**) heating traces; and (**c**) heating traces of the quenched samples.

Figure 6 shows the DSC traces of the (PBT-co-PCT)-b-PTMG copolymers and Table 5 gathers the values of the glass transition temperatures of the soft and hard segments (*T_gs_* and *T_gh_*), crystallization temperature (*T_c_*), melting temperature (*T_m_*) and enthalpies of crystallization (Δ*H_c_*) and fusion (Δ*H_m_*). In Figure 6a, all copolymers crystallize when cooled down from the molten state as they all exhibit a crystallization peak. The crystallization temperature decreases with the increasing *CT* content until 30%; and it increases with the increasing *CT* content when exceeding 30%. The enthalpy of crystallization follows the same trend and reaches a minimum at *M*_PCT_ of around 30%. At around this *CT* content, none of the average sequences of the PBT and PCT hard segments is long enough to fully crystallize.

From the DSC heating traces (Figure 6b), the melting temperature decreases first from 187.8 °C to 150.6 °C and then increases again to 245.2 °C. Meanwhile, the enthalpy of fusion (Δ*H_m_*) and the degree of crystallinity (*X_C_*) follow a trend similar to that of the melting temperature, and reach minima when the *CT* content is around 30%, for the reason above.

Furthermore, Figure 6c shows the glass transition temperature of the (PBT-co-PCT)-b-PTMG copolymers previously quenched from the melt. Their value is estimated through the tangent midpoint of the curves. The glass transition temperature of the soft segments PTMG (*T_gs_*) appears in the vicinity of −14 °C, which is slightly higher than that of PTMG homopolymer because of the loss of free volume of the amorphous domains with the interference of the amorphous hard segments. On the other hand, the glass transition temperature of the hard segments PBT and PCT (*T_gh_*) decreased with increasing CT content until 30%. It is 33.8 °C for PCT0 and 23.5 °C for PCT30. This is due to the structure regularity of the hard segments. When the CT content is less than 30%, *T_gh_* is lower than that of PBT homopolymer because of the influence of the CT content in amorphous. Thereafter, when the CT content is above 30%, the structure of hard segments become regular again, *T_gh_* increases with increasing CT content and reaches 45.5 °C for PCT70.

### 3.5. TGA Analysis

**Figure 7:**
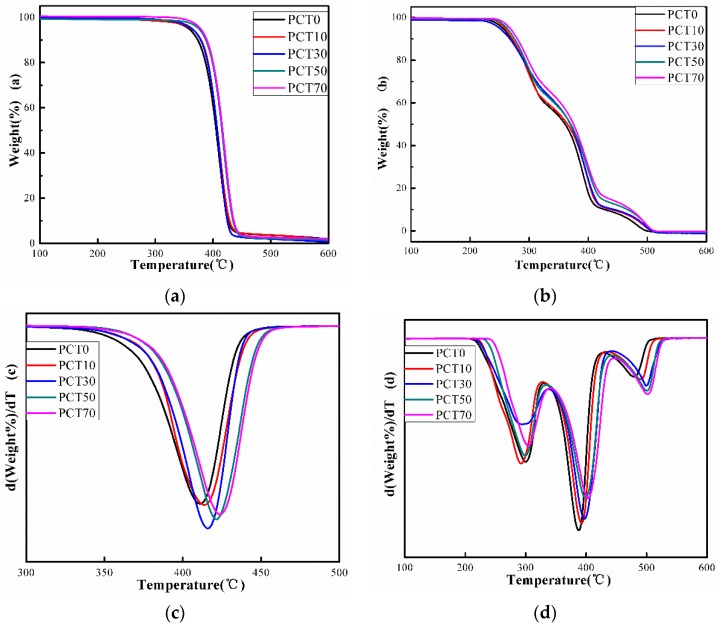
TGA and DTG thermograms of the (PBT-co-PCT)-b-PTMG copolymers at 20 °C min^–^^1^: (**a**) TGA in nitrogen atmosphere; (**b**) TGA in oxygen atmosphere; (**c**) DTG in nitrogen atmosphere; and (**d**) DTG in oxygen atmosphere.

Figure 7 shows the thermal gravimetrical traces of the (PBT-co-PCT)-b-PTMG copolymers. In Figure 7a,c, the thermal decomposition temperature increases with increasing CT content under nitrogen atmosphere, indicating that the thermal stability of the copolymer is improved by CT moieties. This is due to the increase of thermally stable cyclohexylene group [15].

Compared with the thermal decomposition under nitrogen atmosphere, three degradation platforms are observed under the oxygen atmosphere in Figure 7b,d. The first and second platforms are assigned to the oxygen degradation of the ether bond in the soft segment and the copolyester in the hard segments, respectively [42,43]. It is worth noting that the CT moieties mainly increase the thermal stability of the hard segments of the copolymers. The third platform is due to the further oxidation of the oxidation products of the first and second stages.

### 3.6. Mechanical Properties

**Figure 8:**
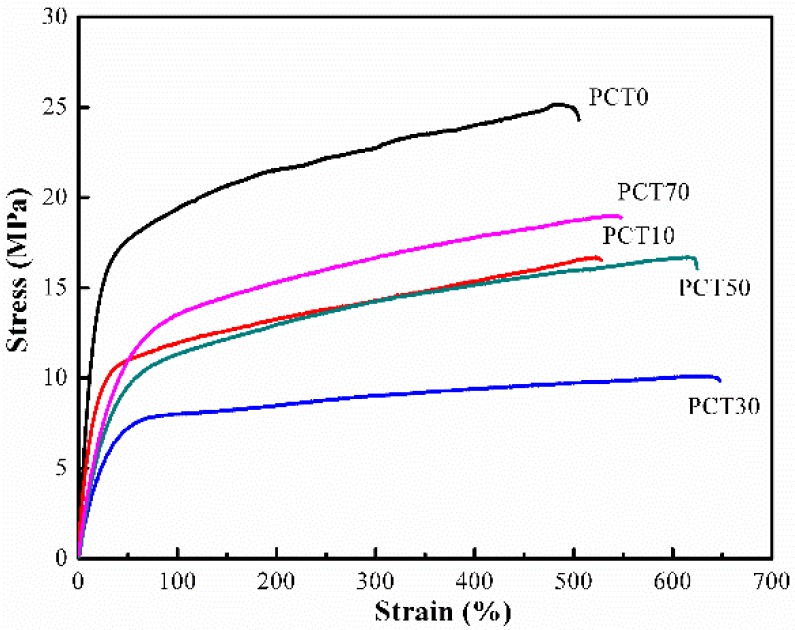
Tensile stress–strain curves of the (PBT-co-PCT)-b-PTMG copolymer.

Figure 8 shows the stress–strain curves of the (PBT-co-PCT)-b-PTMG copolymers. Each curve is the average of five specimens. They all exhibit a typical thermoplastic elastomer behavior: an elastic deformation with small elongations, followed by a decrease in the slope without an apparent yield point. Moreover, the mechanical properties of a (PBT-co-PCT)-b-PTMG copolymer depends very much on its composition, especially the crystallinity of its hard segments. The elastic modulus (E) and tensile strength (σ*_b_*) of PCT30 are the lowest while its elongation at break is the highest, because its crystallinity is the lowest. Table 6 gathers the values of these properties for the (PBT-co-PCT)-b-PTMG copolymers synthesized in this work.

### 3.7. Optical Properties

**Figure 9:**
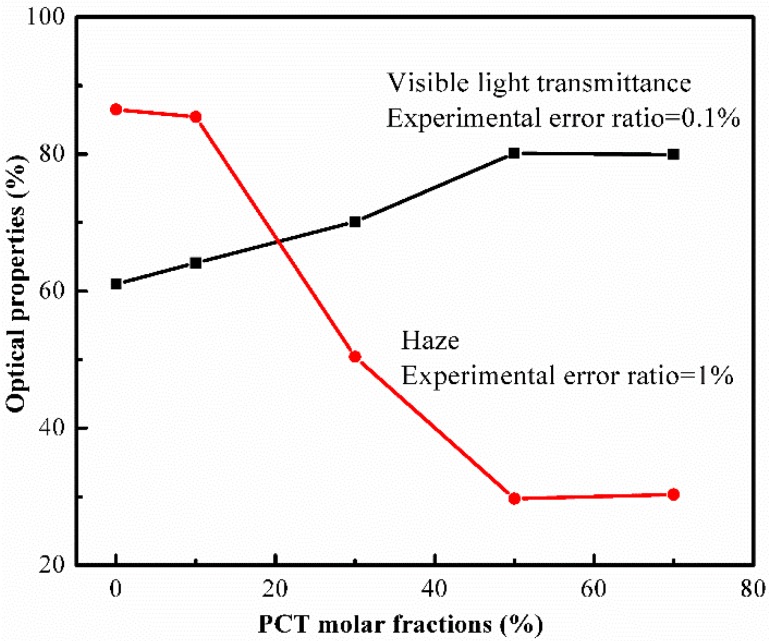
The optical properties of the (PBT-co-PCT)-b-PTMG copolymers.

Figure 9 shows the visible light transmittance and haze of the (PBT-co-PCT)-b-PTMG copolymers as a function of their CT content. Overall, the visible light transmittance increases first and then levels off with the increasing CT content, and concomitantly the haze decreases first and then levels off with the increasing CT content. In other words, the incorporation of CT moieties improves the optical properties of the (PBT-co-PCT)-b-PTMG copolymer.

## 4. Conclusions

In summary, this work shows that partial substitution of the BT hard segments of PBT-b-PTMG copolymers by CT moieties decreases the average sequence length of BT moiety and the crystallinity of the copolymers, resulting in a decrease in elastic modulus and tensile strength, and an increase in the average sequence length of CT moiety and elongation at break. On the other hand, it improves their thermal stability and optical properties.

## Figures and Tables

**Table 1:** Compositions for the synthesis of the (PBT-co-PCT)-b-PTMG copolymers.

Samples	DMT (mol)	CHDM (mol)	BDO (mol)	PTMG (g)
PCT0	0.5	-	0.7	100
PCT10	0.5	0.052	0.648	100
PCT30	0.5	0.153	0.547	100
PCT50	0.5	0.255	0.445	100
PCT70	0.5	0.355	0.345	100

**Table 2:** Compositions, molecular weights and polydispersity of the (PBT-co-PCT)-b-PTMG copolymers.

Samples	*M_PTMG_* (%)	*M_PBT_* (%)	*M_PCT_* (%)	*trans*/*cis*	*M*_n_	*M*_w_	PDI
PCT0	20.2	79.1	-	-	1.8 × 10^4^	3.7 × 10^4^	2.08
PCT10	19.8	69.6	10.6	69.8/30.2	1.7 × 10^4^	3.6 × 10^4^	2.12
PCT30	19.7	49.5	30.8	70.3/29.7	1.7 × 10^4^	3.5 × 10^4^	2.11
PCT50	20.1	28	51.9	71.6/28.4	1.6 × 10^4^	3.3 × 10^4^	2.07
PCT70	20.3	7.6	72.1	69.1/30.9	1.5 × 10^4^	3.1 × 10^4^	2.06

**Table 3:** Sequence distribution analysis of the (PBT-co-PCT)-b-PTMG copolymers.

Samples	Fraction of Dyad			Molar Fraction of Diester		Average Sequence Length		Degree of Randomness
	*f_BB_*	*f_BC_* + *f_CB_*	*f_CC_*	*P_B_*	*P_C_*	*L_nB_*	*L_nC_*	*DR*
PCT0	-	-	-	-	-	-	-	-
PCT10	0.578	0.382	0.04	0.778	0.222	4.1	1.2	1.05
PCT30	0.456	0.441	0.103	0.677	0.323	3.1	1.5	1.01
PCT50	0.192	0.483	0.325	0.434	0.566	1.8	2.3	0.98
PCT70	0.112	0.462	0.426	0.343	0.657	1.5	2.8	1.02

**Table 4:** WAXD data of the (PBT-co-PCT)-b-PTMG copolymers.

Samples	Crystal Plane	2θ (°)	*d* (Å)	β (°)	*L* (Å)
PCT0	0$\bar{1}$1	16.27	5.46	0.59	151.14
	010	17.39	5.10	0.43	207.69
	$\bar{1}$11	20.65	4.29	0.96	93.47
	100	23.33	3.80	0.61	147.77
	1$\bar{1}$1	25.20	3.52	0.64	141.34
PCT10	0$\bar{1}$1	16.06	5.51	0.61	146.15
	010	17.25	5.13	0.45	198.42
	$\bar{1}$11	20.36	4.35	0.98	91.52
	100	23.19	3.83	0.67	134.50
	1$\bar{1}$1	25.07	3.54	0.66	137.02
PCT30	-	-	-	-	-
	010	16.80	5.27	0.47	189.87
	$\bar{1}$11	20.16	4.40	1.01	88.78
	100	22.96	3.87	0.82	109.8
	-	-	-	-	-
PCT50	011	15.13	5.85	0.53	168.02
	010	16.83	5.27	0.46	193.99
	1$\bar{1}$0	19.36	4.58	0.77	116.31
	100	23.56	3.77	0.66	136.64
	$\bar{1}$11	25.72	3.46	0.66	137.20
PCT70	011	15.17	5.83	0.47	189.49
	010	16.83	5.26	0.45	198.31
	1$\bar{1}$0	19.24	4.61	0.73	122.66
	100	23.39	3.79	0.64	140.86
	$\bar{1}$11	25.50	3.49	0.65	139.24

**Table 5:** Thermal properties of the (PBT-co-PCT)-b-PTMG copolymers obtained from DSC.

Samples	Cooling		Heating				
	*T_c_* (°C)	Δ*H_c_* (J·g^−1^)	*T_gs_* (°C)	*T_gh_* (°C)	*T_m_* (°C)	Δ*H_m_* (J·g^−1^)	*W_C_* (%)
PCT0	147.6	22.6	−16.1	33.8	187.8	22.8	19.9
PCT10	118.1	17.0	−16.7	26.4	158.8	17.7	15.9
PCT30	83.9	12.0	−17.0	23.5	150.6	12.1	10.5
PCT50	147.9	13.7	−11.9	42.9	206.5	14.0	15.0
PCT70	198.0	15.6	−12.7	45.5	245.2	15.7	18.6

**Table 6:** The mechanical properties for the (PBT-co-PCT)-b-PTMG copolymers

Samples	Elastic Modulus (E)	Tensile Strength σ_max_ (MPa)	Elongation at Break ε_b_ (%)
PCT0	101.7 (±9.8)	25.1 (±3.7)	505.3 (±49.8)
PCT10	71.2 (±7.4)	16.6 (±1.8)	527.7 (±47.2)
PCT30	33.2 (±3.6)	10.1 (±1.2)	647.7 (±61.3)
PCT50	35.2 (±3.3)	16.7 (±1.7)	624.4 (±63.7)
PCT70	43.4 (±4.7)	19.0 (±2.1)	547.9 (±50.7)
